# Angiotensin I Converting Enzyme Inhibitory Peptides Derived from Phycobiliproteins of Dulse *Palmaria palmata*

**DOI:** 10.3390/md14020032

**Published:** 2016-02-04

**Authors:** Tomoe Furuta, Yoshikatsu Miyabe, Hajime Yasui, Yasunori Kinoshita, Hideki Kishimura

**Affiliations:** 1 Laboratory of Marine Chemical Resource Development, Graduate School of Fisheries Sciences, Hokkaido University, Hakodate, Hokkaido 041-8611, Japan; furuta-tomoe@hro.or.jp (T.F.); yoshikatsu.miyabe@gmail.com (Y.M.); 2 Laboratory of Humans and the Ocean, Faculty of Fisheries Sciences, Hokkaido University, Hakodate, Hokkaido 041-8611, Japan; hagime@fish.hokudai.ac.jp; 3 Department of Research and Development, Hokkaido Industrial Technology Center, Kikyo 379, Hakodate, Hokkaido 041-0801, Japan; kinoshita@techakodate.or.jp; 4 Laboratory of Marine Chemical Resource Development, Faculty of Fisheries Sciences, Hokkaido University, Hakodate, Hokkaido 041-8611, Japan

**Keywords:** ACE inhibitory activity, antihypertension, dulse, *Palmaria palmata*, phycobiliprotein, primary structure, recombinant protein, red algae

## Abstract

We examined the inhibitory activity of angiotensin I converting enzyme (ACE) in protein hydrolysates from dulse, *Palmaria palmata*. The proteins extracted from dulse were mainly composed of phycoerythrin (PE) followed by phycocyanin (PC) and allophycocyanin (APC). The dulse proteins showed slight ACE inhibitory activity, whereas the inhibitory activity was extremely enhanced by thermolysin hydrolysis. The ACE inhibitory activity of hydrolysates was hardly affected by additional pepsin, trypsin and chymotrypsin treatments. Nine ACE inhibitory peptides (YRD, AGGEY, VYRT, VDHY, IKGHY, LKNPG, LDY, LRY, FEQDWAS) were isolated from the hydrolysates by reversed-phase high-performance liquid chromatography (HPLC), and it was demonstrated that the synthetic peptide LRY (IC_50_: 0.044 μmol) has remarkably high ACE inhibitory activity. Then, we investigated the structural properties of dulse phycobiliproteins to discuss the origin of dulse ACE inhibitory peptides. Each dulse phycobiliprotein possesses α-subunit (Mw: 17,477–17,638) and β-subunit (Mw: 17,455–18,407). The sequences of YRD, AGGEY, VYRT, VDHY, LKNPG and LDY were detected in the primary structure of PE α-subunit, and the LDY also exists in the APC α- and β-subunits. In addition, the LRY sequence was found in the β-subunits of PE, PC and APC. From these results, it was suggested that the dulse ACE inhibitory peptides were derived from phycobiliproteins, especially PE. To make sure the deduction, we carried out additional experiment by using recombinant PE. We expressed the recombinant α- and β-subunits of PE (rPEα and rPEβ, respectively), and then prepared their peptides by thermolysin hydrolysis. As a result, these peptides showed high ACE inhibitory activities (rPEα: 94.4%; rPEβ: 87.0%). Therefore, we concluded that the original proteins of dulse ACE inhibitory peptides were phycobiliproteins.

## 1. Introduction

Angiotensin I converting enzyme (ACE: EC 3.4.15.1) is physiologically important in the regulation of blood pressure catalyzing the production of angiotensin II and the destruction of bradykinin [1]. The specific inhibitors of the enzyme therefore have been recognized as effective antihypertensive drugs, but these synthetic ACE inhibitors can cause many significant undesirable side effects [2]. Therefore, the natural safe compounds are desirable for prevention of hypertension. Up to now, ACE inhibitory peptides have been found in enzymatic hydrolysates of many foodstuffs [2,3,4,5,6,7]. However, there are few studies on ACE inhibitory activity and antihypertensive activity of peptides derived from marine algae, especially from red algae. In 2002, Sato *et al.* studied ACE inhibitory peptides from brown alga *Undaria pinnatifida* (wakame) that is a popular traditional foodstuff in Japan [8]. They isolated seven ACE inhibitory peptides from the hydrolysates of *U. pinnatifida* by Protease S “Amano”. Suetsuna *et al.* also reported the antihypertensive effect of *U. pinnatifida* peptide on blood pressure in spontaneously hypertensive rat (SHR) [9]. They isolated ten dipeptides from the alga by hot water extraction and treated SHRs with the four dipeptides containing the great ACE inhibitory potential. On the other hand, Cha *et al.* screened ACE inhibitory activities of methanol and aqueous extracts prepared from twenty-six red algae at the coast of Jeju Iland in Korea [10]. Among aqueous extracts at 20 °C of red algae, *Lomentaria catenata* showed the strongest ACE inhibitory activity and *Lithophyllum okamurae* recorded the second highest activity. Remarkable activities in the methanol extracts at 70 °C were observed in *Grateloupia filicina*, *G. lanceolata* and *Sinkoraena lancifolia*, however no significant activities were found in their aqueous extracts. He *et al.* also measured ACE inhibitory activities of the 48 hydrolysates from marine protein materials [11], and they found the inhibitory activity of protein hydrolysate from red alga *Polysiphonia urceolata* was relatively high. However, they did not study in detail, such as the structure of ACE inhibitory peptides and the original protein of inhibitory peptides.

**Figure 1:**
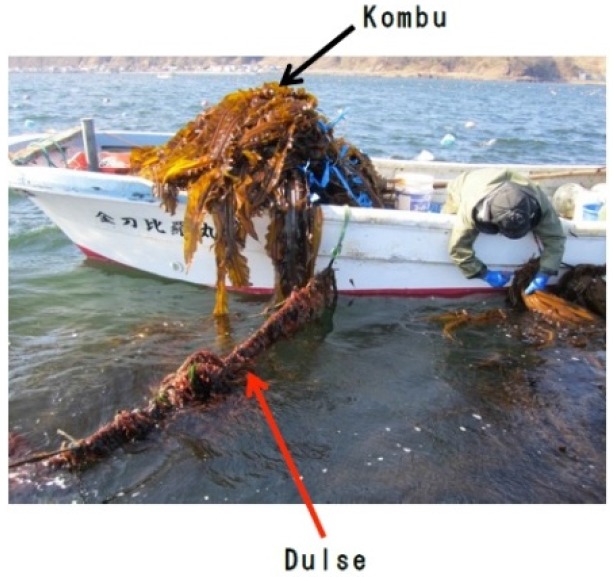
Dulse on the Kombu farming rope.

Dulse (*Palmaria palmata*) is a red alga distributed mainly in high-latitude coastal areas, and is popular in Ireland and Atlantic Canada as a food and a source of minerals. Recently, it was reported the dulse protein hydrolysates show inhibitory effects for renin [12] and dipeptidyl peptidase IV [13]. In Japan, it is rarely eaten and is even removed from Kombu (*Laminaria* sp.) farming areas in Hokkaido Prefecture, because it slows the growth of Kombu (Figure 1). We have recently begun exploring the health benefits of dulse to advance its use as a functional food material. In this study, we examined the ACE inhibitory activity in protein hydrolysates from dulse, and we identified the ACE inhibitory peptides in it. In addition, we investigated the structural properties of dulse phycobiliproteins to discuss the origin of dulse ACE inhibitory peptides.

## 2. Results and Discussion

### 2.1. ACE Inhibitory Activity of Dulse Protein Hydrolysates

**Figure 2:**
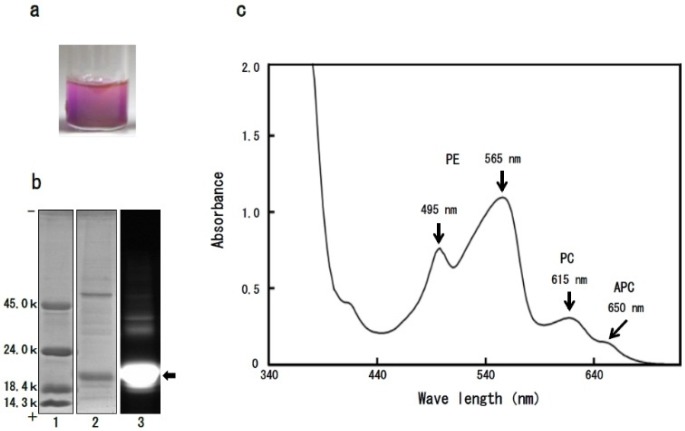
Biochemical properties of dulse proteins. (**a**) Dulse protein extracts; (**b**) Sodium dodecyl sulfate-polyacrylamide gel electrophoresis (SDS-PAGE) of dulse proteins. (1) Molecular weight marker; (2) dulse proteins (CBB R-250 staining); (3) dulse proteins (Fluorescent photography). The arrow indicates α- and β-subunits of phycobiliproteins; (**c**) Visible ray absorption spectra of dulse proteins. The arrows indicate maximal absorption peaks of phycoerythrin (PE: 495 nm and 565 nm), phycocyanin (PC: 615 nm) and allophycocyanin (APC: 650 nm).

Phycobiliproteins play a role of light capturing on photosynthesis in red algae, and the prominent class of them are phycoerythrin (PE), phycocyanin (PC) and allophycocyanin (APC) that are divided on their spectral properties (λ-max of PE: 490–570 nm; λ-max of PC: 610–625 nm; λ-max of APC: 650–660 nm) [14,15,16]. As shown in Figure 2a, the protein extracts from dulse powder assumed bright red colour, which is supposed to originate from PE. The main protein component in extracts possessed molecular weight of about 20,000, and it fluoresced by irradiation of excitation light (490–560 nm) (Figure 2b). In addition, the maximum absorption wavelength of dulse proteins was in the range of 495–565 nm (Figure 2c). The high absorption at PE absorption wavelength demonstrates that the protein extracts are rich in PE.

**Figure 3:**
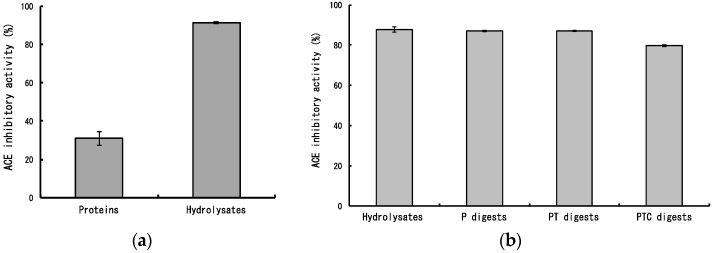
Angiotensin I converting enzyme (ACE) inhibitory activities of dulse hydrolysates and its protease digests. (**a**) ACE inhibitory activities of dulse proteins and its hydrolysates. Proteins: Dulse proteins; Hydrolysates: Dulse hydrolysates; (**b**) ACE inhibitory activities of protease digests. Hydrolysates: Dulse hydrolysates, P: Pepsin digests, PT: Pepsin-trypsin digests, PTC: Pepsin-trypsin-chymotrypsin digests.

Then, we measured ACE inhibitory activity of dulse proteins and its hydrolysates. The dulse proteins indicated slight ACE inhibitory activity, whereas the inhibitory activity was extremely enhanced by thermolysin hydrolysis (Figure 3a). The ACE inhibitory activity of dulse hydrolysates (88%) was hardly affected by additional pepsin (87%), pepsin-trypsin (87%) and pepsin-trypsin-chymotrypsin (80%) digestions (Figure 3b). Therefore, it is suggested that the ACE inhibitory peptides are effectively derived from phycobiliproteins by thermolysin hydrolysis and these peptides may reach the small intestine maintaining the activities.

### 2.2. Isolation of Dulse ACE Inhibitory Peptides

**Figure 4:**
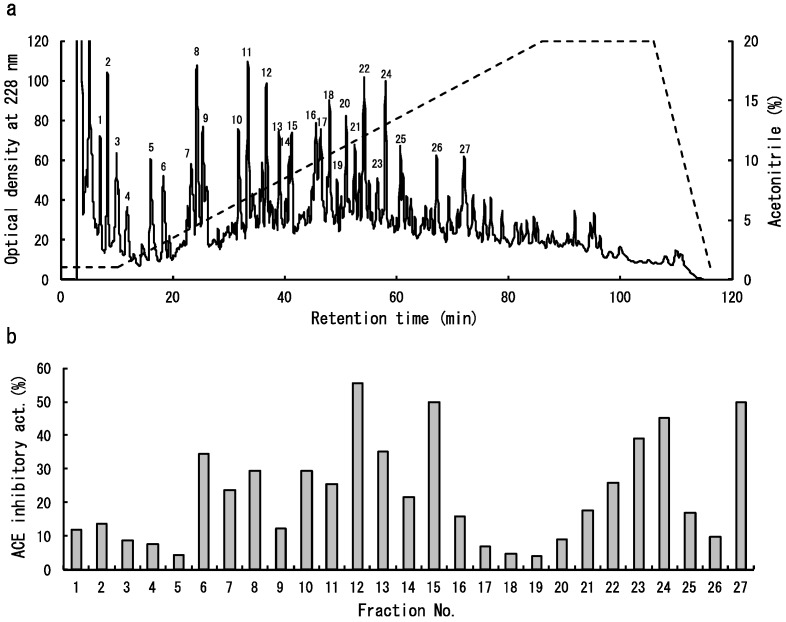
Isolation of ACE inhibitory peptides from dulse protein hydrolysates. (**a**) Separation of peptides on Mightysil RP-18GP column. The peaks marked 1–27 indicate pooled fractions; (**b**) ACE inhibitory activity of each marked fraction.

The dulse protein hydrolysates were separated by reversed-phase high-performance liquid chromatography (HPLC) (Figure 4a). The eluted fractions, named 1–27, were pooled and subjected to ACE inhibitory assay. Relatively high inhibitory activities were detected in several fractions (No. 6, 7, 8, 10, 11, 12, 13, 15, 23, 24 and 27) (Figure 4b). So, the amino acid sequences of peptides in these fractions were analysed. As shown in Table 1, nine peptide sequences of YRD, AGGEY, VYRT, VDHY, IKGHY, LKNPG, LDY, LRY and FEQDWAS were determined. It is well known that the peptides containing hydrophobic amino acid residues with aromatic (Y, F, W) or branched (L, I, V) side chains possess a strong ACE inhibitory effect [17]. All peptides identified in this study also contained the above hydrophobic amino acid residues. Then, four peptides (VYRT, LDY, LRY, FEQDWAS), which were presumed to inhibit ACE effectively, were synthesized and their inhibitory activities were measured. As a result, it was demonstrated that the synthetic LRY (IC_50_: 0.044 μmol) has remarkably high activity, followed by VYRT (IC_50_: 0.14 μmol) (Table 2). Although the IC_50_ value of synthetic LRY is higher than that of chum salmon muscle peptide (IW) [18], it is equivalent to that of sesame peptide (LVY) [19] which is used as antihypertensive agent for the beverage of a food for specified health uses (FOSHU) in Japan. Therefore, it is suggested that the dulse may have a potential as functional foodstuff.

**Table 1:** Analytical sequences of the isolated peptides by reversed-phase high-performance liquid chromatography (HPLC).

Fraction No.	Amino Acid Sequence
6	Not determined
7	Not determined
8	YRD
10	AGGEY
11	Not determined
12	VYRT
13	VDHY
15	IKGHY
	LKNPG
23	Not determined
24	LDY
	LRY
27	FEQDWAS

**Table 2:** ACE inhibitory activities of synthetic peptides.

Amino Acid Sequence	IC_50_ Value (μmol)
Dulse (in this study)	
VYRT	0.14
LDY	6.1
LRY	0.044
FEQDWAS	>2.8
Chum salmon	
IW	0.024 *
Sesame	
LVY	0.045 **

*: cited from the reference [18] after modification of the unit; **: cited from the reference [19] after modification of the unit.

### 2.3. Structure-Function Relationship of Dulse Phycobiliproteins

As described in the preceding section, nine ACE inhibitory peptides were isolated from the dulse protein hydrolysates. Next, we investigated the structural properties of dulse phycobiliproteins to discuss the origin of dulse ACE inhibitory peptides.

**Figure 5:**
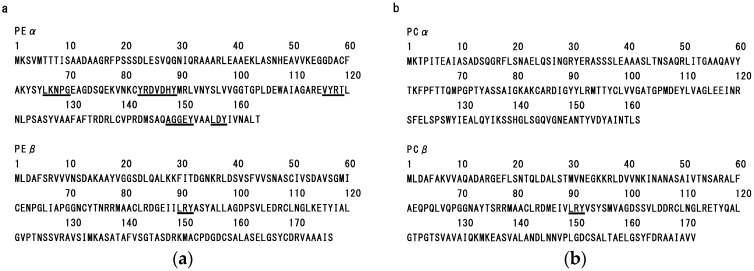

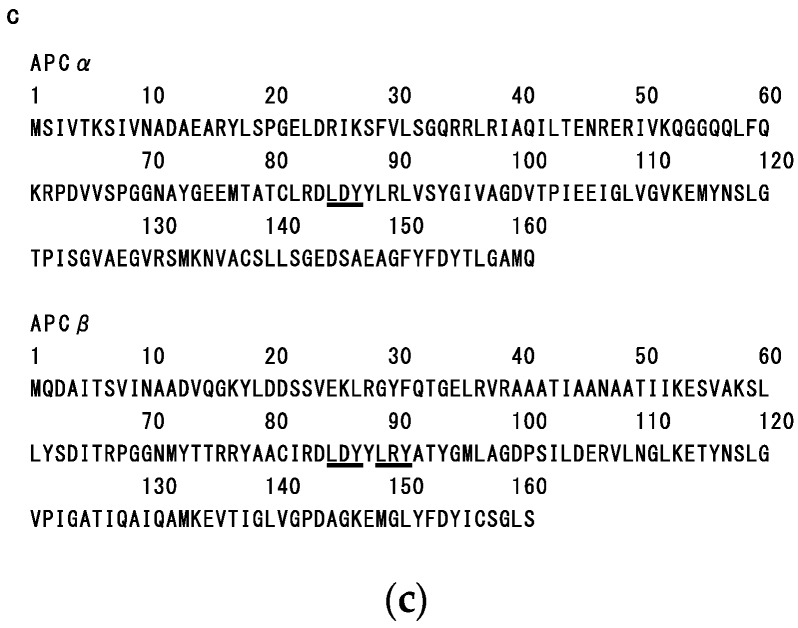
Amino acid sequences of dulse phycobiliproteins. (**a**) Dulse phycoerythrin (GenBank accession number AB625450).PEα: Dulse phycoerythrin α-subunit, PEβ: Dulse phycoerythrin β-subunit; (**b**) Dulse phycocyanin (GenBank accession number AB679831).PCα: Dulse phycocyanin α-subunit, PCβ: Dulse phycocyanin β-subunit; (**c**) Dulse allophycocyanin (GenBank accession number AB742300).APCα: Dulse allophycocyanin α-subunit, PCβ: Dulse allophycocyanin β-subunit.Underlines indicate the sequences of dulse ACE inhibitory peptides.

As shown in Figure 4, the primary structures of dulse PE (GenBank accession number AB625450) (Figure 5a), PC (GenBank accession number AB679831) (Figure 5b) and APC (GenBank accession number AB742300) (Figure 5c) were determined by cDNA cloning method. Each dulse phycobiliprotein possesses α-subunit (Mw: 17,477–17,638) and β-subunit (Mw: 17,455–18,407). Then, we calculated the contents of hydrophobic amino acid residues in dulse phycobiliproteins. As a result, it was clarified that the dulse phycobiliproteins are rich in hydrophobic amino acid residues (PE: 51.0%; PC: 49.1%; APC: 50.9%). Especially, the rates of hydrophobic amino acid residues with aromatic (PEα: 8.5%; PEβ: 5.6%; PCα: 9.9%; PCβ: 5.2%; APCα: 7.5%; APCβ: 8.7%) and branched (PEα: 18.3%; PEβ: 22.0%; PCα: 17.9%; PCβ: 23.3%; APCα: 24.2%; APCβ: 23.0%) side chains are relatively high. Moreover, as shown in Figure 5a, the sequences of LKNPG (amino acid residues 66–70), YRD (83–85), VDHY (86–89), VYRT (116–119), AGGEY (148–152) and LDY (156–158) were detected in the primary structure of PEα, and the LDY also exsists in the APCα (amino acid residues 85–87) and APCβ (85–87) (Figure 5c). The LRY sequence was found in the β-subunits of PE (amino acid residues 90–92), PC (90–92) and APC (89–91) (Figure 5a–c). From these results, it was suggested that the dulse ACE inhibitory peptides were derived from phycobiliproteins, especially PE.

**Figure 6:**
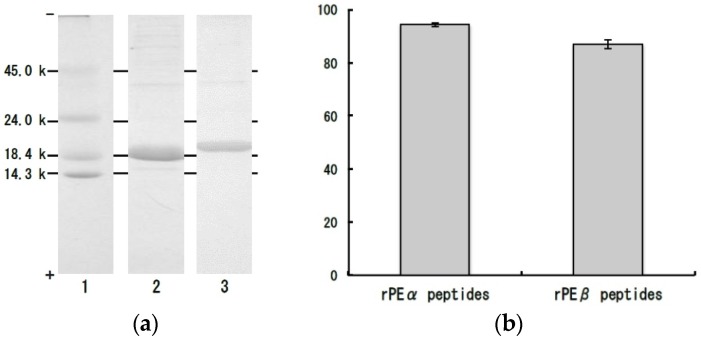
SDS-PAGE of the peptides prepared from recombinant α- and β-subunits of dulse phycoerythrin and ACE inhibitory activities of their peptides. (**a**) SDS-PAGE of recombinant α- and β-subunits of dulse phycoerythrin (CBB R-250 staining). (1) Molecular weight marker, (2) recombinant α-subunits of dulse phycoerythrin, (3) recombinant β-subunits of dulse phycoerythrin; (**b**) ACE inhibitory activities of the peptides prepared from recombinant α- and β-subunits of dulse phycoerythrin. rPEα peptides: The peptides prepared from recombinant α-subunit of dulse phycoerythrin. rPEβ peptides: The peptides prepared from recombinant β-subunit of dulse phycoerythrin.

To make sure the deduction, we carried out additional experiment by using recombinant PE. We expressed the recombinant α- and β-subunits of PE (rPEα and rPEβ, respectively) in *Escherichia coli* Rosetta 2 (DE3) (Figure 6a), and then we prepared their peptides by thermolysin hydrolysis. As described in Figure 6b, the peptides prepared from rPEα (94.4%) and rPEβ (87.0%) also strongly inhibited ACE activities. In addition, the rPEα and rPEβ are apo-proteins and they lack the chromophores. The result also indicates the main substance of ACE inhibition is peptide derived from dulse PE apo-protein. Therefore, we concluded that the original proteins of dulse ACE inhibitory peptides are phycobiliproteins.

## 3. Experimental Section

### 3.1. Materials

Dulse was harvested off Usujiri, Hokkaido, Japan in February, and the samples were stored at −20 °C until use.

ACE from rabbit lung was purchased from Sigma Chemical Co. (St. Louis, MO, USA). Hyppuryl-l-histidyl-l-leucine (Hip-His-Leu), thermolysin (EC 3.4.24.27) from *Bacillus thermoproteolyticus*, pepsin (EC 3.4.23.1) from porcine stomach, trypsin (EC 3.4.21.4) from bovine pancreas and chymotrypsin (EC 3.4.21.1) from bovine pancreas were purchased from Wako Pure Chemical (Osaka, Japan). Synthetic peptides (purity: 99.4%–99.8%) were purchased from Medical Biological Laboratories Co. (Nagoya, Japan). All other reagents were purchased from Wako Pure Chemical Industries (Osaka, Japan).

### 3.2. Preparation of Protein Hydrolysates from Dulse

The frozen samples were lyophilized and ground into a fine powder by Wonder Blender WB-1 (OSAKA CHEMICAL Co., Osaka, Japan). Proteins were extracted from the powder by adding 20 *v*/*w* of distilled water at 4 °C for 7 h. The extract was centrifuged (H-200, Kokusan, Tokyo, Japan) at 4 °C, 15,000 *g* for 10 min, and then the supernatant was used as dulse proteins. The dulse proteins were hydrolyzed by 1.0 wt % of thermolysin at 70 °C for 3 h, and the reaction was ended by heat treatment at 100 °C for 5 min. Subsequently, the solution was centrifuged at 4 °C, 15,000 *g* for 10 min. The supernatant was dried by lyophilisation into dulse hydrolysates.

The dulse hydrolysates were dissolved in distilled water and the solution was adjusted to pH 2.0. The hydrolysates were digested by 1.0 wt % of pepsin at 37 °C for 3 h (pepsin digests). After the reaction, the pepsin digests were adjusted to pH 8.0. Then, 1.0 wt % of trypsin was added to the pepsin digest and the mixture was incubated at 37 °C for 3 h (pepsin-trypsin digests). Subsequently, the pepsin-trypsin digests were treated by 1.0 wt % of chymotrypsin at 37 °C for 3 h (pepsin-trypsin-chymotrypsin digests). The digests were boiled for 5 min to inactivate the enzymes, and then centrifuged at 4 °C, 15,000 *g* for 10 min. The supernatants were dried by lyophilisation.

### 3.3. Isolation of Peptides from Dulse Protein Hydrolysates

The dulse hydrolysates were dissolved in ultra pure water containing 0.1% trifluoroacetic acid (TFA) and applied to sequential filtration by Millex-GV (pore size: 0.22 μm) and Millex-LG (pore size: 0.20 μm). Peptides in the filtrate were isolated by reversed-phase HPLC with a Mightysil RP-18GP column (4.6 × 150 mm) (Kanto Kagaku, Tokyo, Japan) using a linear gradient of acetonitrile (1%–20%) containing 0.1% TFA at a flow rate of 1.0 mL/min.

### 3.4. ACE Inhibitory Assay

ACE inhibitory assay was carried out according to the method of Cheng and Cushman [20] with some modifications. Fifteen microliters of sample solution (5.0 mg/mL) were added to 30 μL of ACE (0.2 units/mL), and the mixture was pre-incubated at 37 °C for 5 min. Thirty microliters of Hip-His-Leu solution (12.5 mM in 0.1 M sodium borate buffer containing 400 mM NaCl at pH 8.3) were added to the mixture. After incubation at 37 °C for 1 h, the reaction was stopped by adding 75 μL of 1.0 M HCl. The released hippuric acid was extracted with 450 μL of ethyl acetate. Four hundred microliters of the upper layer were evaporated, and then the hippuric acid was dissolved in 1.5 mL of distilled water. The absorbance at 228 nm of the solution was measured by a spectrophotometer. The inhibition was calculated from the equation [1 − (As − Asb)/(Ac − Acb)] × 100, where Ac is the absorbance of the buffer, Acb is the absorbance when the stop solution was added to the buffer before the reaction, As is the absorbance of the sample, and Asb is the absorbance when the stop solution was added to the sample before the reaction. In this study, we defined the IC_50_ as an absolute quantity of peptide to inhibit 50% of 1.0 U ACE.

### 3.5. Analysis of Visible Ray Absorption Spectrum of Dulse Proteins

The visible ray absorption spectrum of dulse protein extracts analyzed by a spectrophotometer (U-1800: HITACHI, Tokyo, Japan).

### 3.6. Polyacrylamide Gel Electrophoresis of Dulse Proteins

Sodium dodecyl sulfate-polyacrylamide gel electrophoresis (SDS-PAGE) was carried out using a 0.1% SDS-13.75% polyacrylamide slab-gel by the method of Laemmli (1970) [21]. The gel was stained with 0.1% Coomassie Brilliant Blue (CBB) R-250 in 50% methanol-7% acetic acid and the background of the gel was destained with 7% acetic acid. Fluorescence of phycobiliprotein on slab-gel was detected by gel documentation LED illuminator (VISIRAYS AE-6935GN: ATTO, Tokyo, Japan).

### 3.7. Analysis of Amino Acid Sequences of Dulse ACE Inhibitory Peptides

The amino acid sequences of dulse ACE inhibitory peptides were analyzed by Edman degradation method using a protein sequencer (Procise 492HT: Perkin Elmer, Waltham, MA, USA) and Matrix Assisted Laser Desorption/Ionization Time Of Flight Tandem Mass Spectrometry (MALDI-TOF/MS/MS) method using a 4700 Proteomics Analyzer with Denovo Explorer software (Applied Biosystems, Carlsbad, CA, USA).

### 3.8. Bacterial Expression of Recombinant α- and β-Subunits of Dulse Phycoerythrin

Bacterial expression of recombinant α- and β-subunits of dulse PE was carried out according to the method of previous paper [22] with some modifications. The 5′- and 3′-terminal nucleotide sequences of the cDNAs encoding α- and β-subunits of dulse PE (GenBank accession number AB625450) were modified by polymerase chain reaction (PCR) with two sets of primers as shown in Table 3 (α-subunits: RDPEαF and RDPEαR, β-subunits: RDPEβF and RDPEβR). By the PCR, an *Nco* I site which includes an ATG sequence being applicable to a translational start codon was introduced to 5′-terminus of each cDNA, while a *Bam*H I site was introduced to 3′-terminal. The modified cDNAs encoding α- and β-subunits of dulse PE were digested with *Nco* I and *Bam*H I and were inserted into the expression plasmid pET-16b (Novagen, Madison, WI, USA). The recombinant subunits (rPEα and rPEβ) were expressed in *Escherichia coli* Rosetta 2 (DE3) (Novagen, Madison, WI, USA) by induction with isopropyl-β-D(−)-thiogalactopyranoside (Wako Pure Chemical Industries, Osaka, Japan). The rPEα and rPEβ produced as inclusion bodies were dissociated with 8 M urea and 10 mM 2-mercaptoethanol. The dissolved solutions were centrifuged at 4 °C, 10,000 *g* for 10 min and the supernatants were renatured by dialyzing against 10 mM Tris-HCl buffer (pH 8.0) into dulse rPEα and rPEβ (Figure 6a).

**Table 3:** Primers for expression of recombinant phycoerythrin (PE).

Primer	Sequence (5′→3′)
RDPEαF	GGAGATTACCATGGAATCAG
RDPEαR	AAGGGATCATTAGGTTAAAG
RDPEβF	TAAGGAGAGTTBCATGGTTG
RDPEβR	AACAGGATCCATTAGCTAATTGCAGC
